# An analysis of financial protection and financing incidence of out-of-pocket health expenditures in Kazakhstan from 2018 to 2021

**DOI:** 10.1038/s41598-024-59742-9

**Published:** 2024-04-17

**Authors:** Askhat Shaltynov, Yulia Semenova, Madina Abenova, Assel Baibussinova, Ulzhan Jamedinova, Ayan Myssayev

**Affiliations:** 1 Epidemiology and Biostatistics Department, Semey Medical University, Semey, Kazakhstan; 2 School of Medicine, Nazarbayev University, Astana, Kazakhstan; 3 Department of the Science and Human Resources, Ministry of Healthcare of the Republic of Kazakhstan, Astana, Kazakhstan

**Keywords:** Health policy, Health care

## Abstract

Universal health coverage relies on providing essential medical services and shielding individuals from financial risks. Our study assesses the progressivity of out-of-pocket (OOP) payments, identifies factors contributing to healthcare expenditure inequality, and examines catastrophic health expenditures (CHE) prevalence in Kazakhstan from 2018 to 2021. Using retrospective analysis of National Statistics Bureau data, we employed STATA 13 version for calculations CHE incidence, progressivity, Lorenz and concentration curves. In 2020–2021, OOP expenditures in Kazakhstan decreased, reflecting a nearly twofold reduction in the CHE incidence to 1.32% and 1.24%, respectively. However, during these years, we observe a transition towards a positive trend in the Kakwani index to 0.003 and 0.005, respectively, which may be explained by household size and education level factors. Increased state financing and quarantine measures contributed to reduced OOP payments. Despite a low healthcare expenditure share in gross domestic product, Kazakhstan exhibits a relatively high private healthcare spending proportion. The low CHE incidence and proportional expenditure system suggest private payments do not significantly impact financial resilience, prompting considerations about the role of government funding and social health insurance in the financing structure.

## Introduction

UHC is achieved when individuals genuinely receive the necessary medical services and are protected from financial risks^1^.

Financial protection is fundamental to UHC and constitutes one of its ultimate goals. Healthcare financing policies directly impact financial protection. Financial protection is achieved when OOP for medical services do not impose financial hardship on individuals and do not threaten their quality of life. Approximately 930 million people (12.7% of the global population) have experienced CHE, allocating at least 10% of their family budget to pay for healthcare services out of their own pockets. Around 90 million people (1.2% of the global population) still live in “extreme poverty” (living on $1.90 per day or less) because they pay for medical care out of their own pockets^2^. The World Health Organization (WHO) and the World Bank propose a set of Sustainable Development Goal (SDG) indicators in their reports to assess financial protection for the population, such as SDG Indicator 3.8.2—the proportion of a country’s population with CHE. CHE serve as a metric for measuring financial hardships when personal healthcare expenses exceed a defined threshold of household affordability^3^.

The prevalence of CHE typically remains at a very low level in countries where the share of OOP in total healthcare expenditures is below 15% or close to 15%. In 2016, according to WHO data, this indicator stood at 36% in Kazakhstan, 40% in the Russian Federation, 58% in Kyrgyzstan, 52% in Uzbekistan, 36% in Belarus, 10% in France, 15% in the United Kingdom, and 16% in Turkey^4^. For comparison, in 2009, according to a WHO report on OOP among former Soviet Union countries, this indicator was 40% for Kazakhstan, 29% for the Russian Federation, 40% for Kyrgyzstan, 52% for Uzbekistan, and only 20% for Belarus^5^. According to the latest data from the National Health Accounts published in Kazakhstan, the share of private expenditures relative to total healthcare expenditures in 2022 was 38%, of which 30.95% constituted household expenditures^6^.

An equally significant factor contributing to the achievement of UHC is the equitable distribution of healthcare expenditures across all income strata of the population^7^. The set of methods employed to assess the fairness of a healthcare financing system is referred to as Financing Incidence Analysis (FIA)^8^. The most straightforward method within FIA is the analysis of structural progressivity, in which households are classified by quantiles of OOP to assess progressivity^9^. Another widely used method for assessing financial inequality in OOP expenditures is the analysis of effective progressivity, calculated using the Kakwani Index^10^.

OOP constitute one of the most crucial financing mechanisms in many healthcare systems, particularly in developing countries. This adversely affects equity and pushes vulnerable population groups into poverty^9^.

In 2018, Kazakhstan’s healthcare spending (2.9% of GDP) lagged behind OECD countries. Since the dissolution of the Soviet Union in 1991, Kazakhstan has struggled with the legacy of the Semashko system, facing ongoing challenges during the transition to a market economy. Recently, Kazakhstan has implemented a mandatory health insurance system, overseen by the Social Health Insurance Fund, which began purchasing publicly paid health services in 2020. The current model is not considered fully insurance-based; rather, it combines elements of both budgetary and insurance funding. Notably, not all residents are covered by this system, as evidenced by the 16.2% of the population who remained uninsured by the end of 2020. OOP spending as a percentage of current healthcare expenditures in Kazakhstan decreased from 33.5 to 31% from 2018 to 2022, while government expenditures increased from 60.9 to 62%^6,11–13^.

The lack of data on progressivity, coupled with a high proportion of OOP payments within the healthcare system, prompted our investigation. Therefore, the aim of our study was to assess the progressivity of OOP payments for healthcare, identify factors influencing the inequality of healthcare OOP payments, and determine the incidence of CHE.

## Methods

### Study design

A retrospective analysis of OOP expenditures on healthcare was conducted based on secondary data provided by the National Statistics Bureau of the Agency for Strategic Planning and Reforms of the Republic of Kazakhstan. The study was conducted in accordance with the Strengthening the Reporting of Observational Studies in Epidemiology (STROBE) reporting guidelines.

### Data sources

National Statistics Bureau of the Agency for Strategic Planning and Reforms of the Republic of Kazakhstan is the authorized governmental body responsible for collecting and analyzing statistical information in the Republic of Kazakhstan, using established and approved statistical forms. For our study, we utilized the statistical form of the nationwide statistical observation, “Quarterly Household Expenditure and Income Questionnaire” (index D004, quarterly frequency), as well as the statistical form of the nationwide statistical observation, “Control Card of Household Composition” (index D008, annual frequency with quarterly refinement)^10^.

The secondary data of households’ income and expenditures consisted of databases in the form of D004—“Quarterly Household Expenditure and Income Questionnaire” for the years 2018–2021 in the Republic of Kazakhstan. According to the instructions, all households participating in the sample survey of household living standards are subject to statistical observation. The questionnaire is completed by the head of the household or the household member who is most involved in managing the household and is knowledgeable about the expenses and incomes of other family members. The form reflects information on the income of each household member aged 15 and older for the current quarter, and one of its subsections contains information on household expenditures on healthcare, including services of specialist doctors in outpatient clinics, primary doctor visits, dental services, services of medical laboratories and X-ray rooms, services of nurses and midwives, specialized paramedical services, sanatorium services, services of general practitioners in hospitals, services of rehabilitation centers, treatment in day hospitals, services of specialist doctors in hospitals, informal healthcare expenses. D008—“Control Card of Household Composition” statistical observation is designed to compile a list of all members of the surveyed household and gather socio-demographic characteristics for each individual. All households participating in the sample survey of households for assessing the standard of living of the population are subject to this statistical observation. The Control Card is completed by an interviewer at the beginning of the year (January), followed by quarterly updates to capture any relevant changes during the quarter. The respondent for the Control Card is the head of the household.

### Sampling methods

The household sample is constructed through a two-stage probabilistic (random) sampling method employing both stratification and random selection procedures at each stage of sampling. Initially, the general population is stratified based on regional distribution, distinguishing between urban and rural areas, resulting in 31 strata. These strata encompass selected urban and rural areas across sixteen regions of the country, with the exception of Astana and Almaty cities, which lack rural areas. Within each stratum, a number of clusters, termed Primary Selection Units (PSUs), are chosen using a probability proportional to size (PPS) approach based on the number of households within the stratum. In the subsequent stage, a certain number of households are selected from each sampled PSU, guided by a list of individual dwellings within the PSU. The dwellings to be surveyed are chosen with equal probability from the eligible dwellings within the PSU. To obtain data representative of the general population, statistical weighting of the survey results is performed. This is achieved by assigning a statistical weight to each surveyed household, reflecting the total number of households represented by the portion that entered the sample. Weighting coefficients for indicators of the population’s living standards are calculated quarterly. Data on the distribution of surveyed households separately by urban and rural populations in a regional breakdown are used to calculate the weights^14,15^.

According to National Statistics Bureau methodology the sample size was 12,000 households every year of study for D004 and D008 surveys: the sample of 400 PSU are allocated to each of the 31 strata according to probability proportional to size; then in each PSU, 30 households are selected in order to reach a total of 400 × 30 = 12,000 households^15^.

From these databases, we extracted information on income, expenditures, socio-demographic characteristics, household size, and the number of children.

The Adult Equivalent Scale (AES) was employed to assess the household size. The AES defines household size in a manner that considers economies of scale and household composition, and is presented in the following Eq. (1):

1 $$AES = \left( {A + \alpha C} \right)^{\theta } ,$$

where *A* is the number of adults in the household, *C* is the number of children, *α* is a measure of the relative weight accorded to children, *θ* is a measure of economies of scale^16^.

In our study, we used *α* = 0.5 and *θ* = 1, as sensitivity analysis on the data from the National Statistics Bureau of the Agency for Strategic Planning and Reforms of the Republic of Kazakhstan did not reveal significant differences in the coefficients *α* and *θ*^17^.

### Catastrophic health expenditure calculations

The two main methods used to measure the incidence of CHE are the budget share approach and the ability to pay approach. Our study used the Budget Share Approach (or baseline approach) used by World Bank researchers^18^. CHE were defined as occurring when OOP exceed a share of 10% and 25% of the total household income. These thresholds were utilized based on the criteria set by the WHO and the World Bank in their reports^19^. Household incomes, rather than expenditures, were utilized as the income measure due to the absence of data on household expenditures.

### Progressivity of OOP health expenditure calculations

To assess inequality in OOP expenditures on healthcare, an analysis of effective progressivity was conducted, calculating the Kakwani Index (K) (2):

2 $$K = C{-}G,$$

where *C* is the concentration index for healthcare expenditures, *G* is the Gini coefficient for household incomes (ability to pay).

Proportional financing, where the Kakwani Index theoretically equals zero, corresponds to a situation where the Lorenz curve and the concentration curve overlap. In some instances, the Kakwani Index may be zero when the two curves intersect. The Kakwani Index ranges from − 2 to 1; negative values indicate a regressive financing system, positive values signify a progressive system, and values close to zero suggest a proportional system^20^.

In turn, the Concentration Index, equal to the absolute value of twice the area between the equality line and the concentration curve, allows the calculation of perceived poor and very poor health using the Eq. (3):

3 $$CI = \frac{2}{\mu }cov\left( {y_{i} ,R_{i} } \right)$$

where *Ri*—is the rank of the individual in the income distribution; *μ* = $\frac{1}{n}{\sum }_{i=1}^{n}{h}_{i}$, where *n*—n is the number of respondents in the sample.

The concentration index ranges from − 1.0 (a situation where the poorest household contributes all health-care payments) to + 1.0 (where all health-care payments are made by the richest household).

The Gini coefficient is calculated using the Brown Eq. (4):

4 $$G = 1 - \mathop \sum \limits_{k = 2}^{n} \left( {X_{k} - X_{k - 1} } \right)\left( {Y_{k} + Y_{k - 1} } \right) \vee$$

And Gini formula (5):

5 $$G = \frac{{\sum \sum y_{i} - y_{j} \vee }}{{2n^{2} \overline{y}}}$$

where G—Gini coefficient; *X*_*k*_—cumulative share of the population (ranked by increasing incomes); *Yk*—the share of income collectively received by *X*_*k*_; *n*—the number of households; *y*_*k*_—the share of household income in the total income; $\overline{y}$—the arithmetic mean of household income shares.

**Figure 1:**
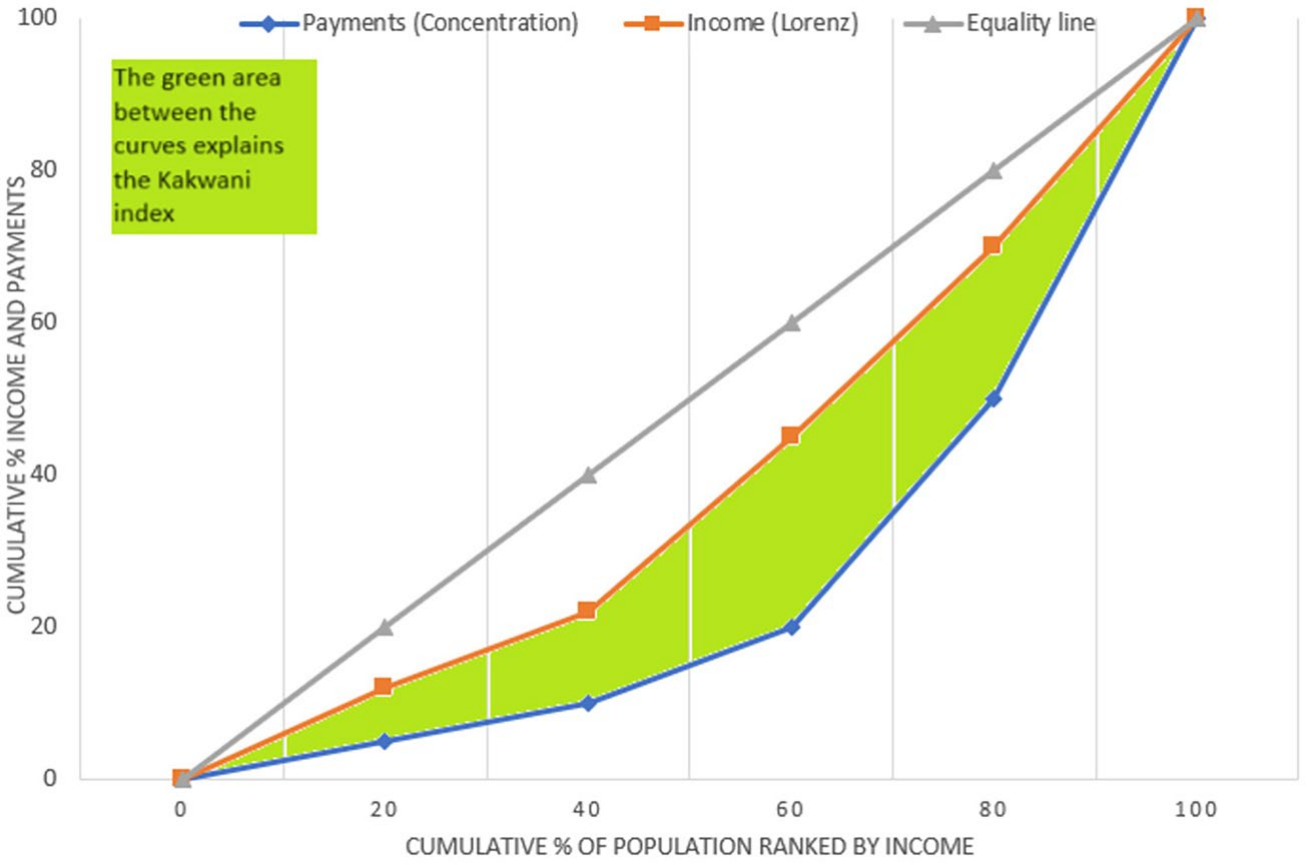
Lorenz and concentration curves explanation.

Concentration curves, utilized for measuring health inequality, have evolved from the Lorenz curve methodology^21,22^. The Lorenz curve represents the graphical depiction of income distribution, where the curve equals a straight diagonal line on a rectangular coordinate system when healthcare resources are evenly distributed. Otherwise, the curve deviates from the diagonal, and the Gini coefficient equals one. The Gini coefficient is a metric that is twice the area between the Lorenz curve and the diagonal line with a zero value in the case of complete equality, and one if all resources are concentrated “in one hand”^23^. For visual understanding and interpretation, we have presented Fig. 1, explaining the Lorenz and Concentration curves.

For analyzing factors influencing the concentration index, the recentered influence function index ordinary least squares (RIF-I-OLS) regression was employed^24^.

### Statistical analysis

Data preparation for analysis was carried out in RStudio version 2023.06.2. The statistical analysis was conducted using STATA version 13 with the utilization of packages -fia-^16^, -fpcata-^25^, -glcurve7-^26^, -rifireg-^24^. The Concentration index, Gini index, and Kakwani index were calculated only for OOP health expenditures on individual level.

### Ethics declarations

The research protocol has been approved by the decision of the institutional review board of Semey Medical University (Protocol #8 dated May 24, 2022), which waived the informed consent as the data were obtained in an unidentifiable manner. This study adhered to the principles depicted in the Declaration of Helsinki.

### Ethics approval and consent to participate

The article relies on secondary data presented in de-identified form and does not necessitate individual informed consents.

## Results

Table 1 presents the characteristics of the sample at the level of individual observations as well as at the household level over the 4 years from 2018 to 2021.

**Table 1:** Sample characteristics.

Sample variables		2018 n = 31,058		2019 n = 30,938		2020 n = 30,962		2021 n = 30,711	
Gender	Male	13.983	45.02%	13.792	44.58%	13.669	44.15%	13.589	44.25%
	Female	17.075	54.98%	17.146	55.42%	17.293	55.85%	17.122	55.75%
Age	15–24	5.347	17.22%	5.135	16.60%	5.016	16.2%	5.139	16.73%
	25–44	11.858	38.18%	11.568	37.39%	11.052	35.7%	11.038	35.94%
	45–59	8.272	26.63%	8.310	26.86%	7.792	25.17%	7.685	25.02%
	60–74	4.496	14.48%	4.890	15.81%	5.955	19.23%	5.755	18.74%
	> 74	1.085	3.49%	1.035	3.35%	1.147	3.7%	1.094	3.56%
Area	Urban	14.498	46.68%	15.626	50.51%	15.869	51.25%	15.618	50.85%
	Rural	16.560	53.32%	15.312	49.49%	15.093	48.75%	15.093	49.15%
Marital status	Married	18,882	60.80%	18.765	60.65%	18.210	58.81%	18.101	58.94%
	Divorced	2.041	6.57%	2.059	6.66%	2.269	7.33%	2.170	7.07%
	Widow	2.925	9.42%	2.987	9.65%	3.383	10.93%	3.279	10.68%
	Single	5.945	19.14%	5.800	18.75%	5.802	18.74%	5.792	18.86%
	N/a	1.265	4.07%	1.327	4.29%	1.289	4.19%	2.170	4.46%
Education	No education	32	0.10%	44	0.14%	49	0.16%	46	0.15%
	Preschool	7	0.02%	6	0.02%	6	0.02%	3	0.01%
	Elementary	955	3.07%	924	2.99%	873	2.82%	882	2.87%
	Basic secondary	3.113	10.02%	3.024	9.77%	3.035	9.8%	2.919	9.50%
	Vocational secondary	19.22	61.88%	19.031	61.51%	18.296	61.13%	18.779	61.15%
	Undergraduate	7.697	24.78%	7.870	25.44%	8.044	25.98%	8.053	26.22%
	Graduate	34	0.11%	39	0.13%	29	0.09%	29	0.09%

Household variables		2018 n = 12,000		2019 n = 12,000		2020 n = 12,000		2021 n = 12,000	
Household size	1	1.324	11.03%	1.366	11.38%	1.556	12.97%	1.571	13.09%
	2	2.270	22.67%	2.739	22.82%	2.803	23.36%	2.798	23.32%
	3–5	6.225	51.88%	6.164	51.37%	5.818	48.48%	5.726	47.72%
	> 5	1.731	14.43%	1.731	14.43%	1.823	15.19%	1.905	15.88%
Number of children under 15 years	1	8.516	70.97%	8.536	71.13%	8.696	72.47%	8.642	72.02%
	2	2.070	17.25%	2.033	16.94%	1.878	15.65%	1.884	15.7%
	3–5	1.396	11.63%	1.413	11.78%	1.405	11.71%	1.456	12.13%
	> 5	18	0.15%	18	0.15%	21	0.17%	18	0.15%

Table 2 presents the key descriptive statistics of healthcare expenditures for the years 2018–2021, both for the overall sample and stratified by income quintiles. These metrics aid in assessing the level of healthcare spending based on income levels, identifying trends in expenditure dynamics, and evaluating the financial burden that the population bears in relation to medical expenses. Elevated values of standard deviation and interquartile range indicate significant variability in expenditures within each quintile. The overall trend indicates that the percentage of income spent on healthcare remains relatively stable, with minor fluctuations over the period. On the whole, average healthcare expenditures per capita decreased in 2020 compared to previous years across all quintiles. This could also be a consequence of changes in healthcare consumption due to the pandemic. The proportion of income spent on healthcare decreased in most quintiles in 2020.

**Table 2:** OOP payments for health services by income quintiles per capita per year.

Year	Income quintile	Mean (in tenge*)	SD (in tenge*)	Median (in tenge*)	IQR (in tenge*)	Proportion (%) of income spent on health services
2018	Q1	16131.75	33575.51	4000	17,400	0.017032
	Q2	23821.35	46564.39	9789.286	29,000	0.014658
	Q3	30491.05	52213.13	12566.67	38,000	0.014041
	Q4	39702.53	62246.04	18,000	44,080	0.013946
	Q5	72693.87	116452.8	32,400	77533.33	0.015532
	Total	36566.42	71253.16	12833.33	40,800	0.014895
2019	Q1	16499.99	35898.99	4320	18,500	0.020903
	Q2	23995.41	42982.87	10,000	28,580	0.017730
	Q3	34542.92	52793.83	16,200	44444.44	0.019805
	Q4	45611.54	70814.11	20933.33	54,000	0.019828
	Q5	68656.26	107097.6	31,875	76013.46	0.018746
	Total	37858.77	69385.31	14,700	43,920	0.019230
2020	Q1	13573.83	32633.25	0	13,800	0.011044
	Q2	22354.38	50196.66	4857.143	25555.56	0.010726
	Q3	27199.94	50191.59	9500	33333.33	0.010655
	Q4	38278.13	89670.17	11111.11	40363.64	0.010567
	Q5	59228.16	119414.8	19833.33	62,000	0.011173
	Total	32123.31	76984.95	7500	33,500	0.010864
2021	Q1	16924.41	42200.39	0	16,000	0.012372
	Q2	26778.73	62337.1	5000	31,200	0.011710
	Q3	33331.53	65538.88	8338	40,000	0.011140
	Q4	47467.97	96614.62	13,333	53,066	0.012048
	Q5	75566.9	141964.7	24,000	80,000	0.012143
	Total	40048.19	91,125	8000	41,333	0.011900

*1 USD = 345 tenge (2018), 1 USD = 383 tenge (2019), 1 USD = 414 tenge (2020), 1 USD = 426 tenge (2021).

**Figure 2:**
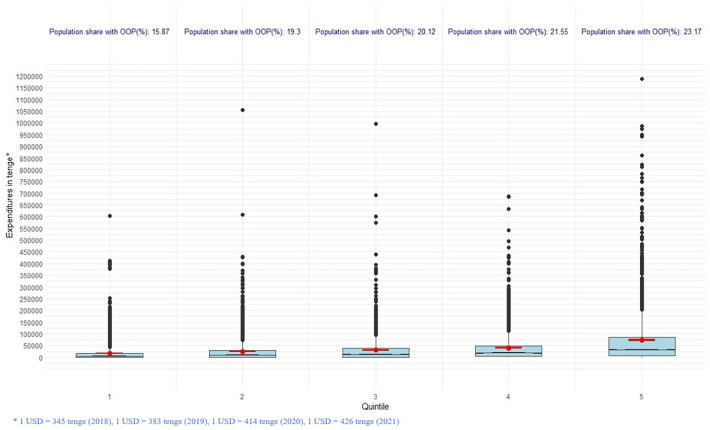
OOP expenditure distribution and population share.

The distribution of healthcare expenditures across quintiles, along with the respective proportion of individuals in each quintile incurring OOP expenses for healthcare, is depicted in Fig. 2. It is noted that as the income quintile increases, there is an increase in the proportion of individuals incurring OOP healthcare expenses. This is likely associated with the fact that less affluent segments of the population refrain from spending money on healthcare.

The occurrence of CHE, considering both the 10%- and 25%-income thresholds, is detailed in Table 3. Findings for the 10% threshold reveal that the percentage, both across all categories and within quintiles, peaked in 2019 before experiencing a notable decline in 2020. Notably, the incidence is significantly pronounced in the 1st quintile, with statistically significant negative concentration index values observed for both the 1st quintile and the entire sample. This indicates a concentration of CHE among the least economically advantaged quintile of the population.

**Table 3:** Incidence of catastrophic payments.

Year	Level	Threshold	Incidence (%)	10% threshold concentration index (*p* value)	Threshold	Incidence (%)	25% threshold concentration index (*p* value)
2018	Total	10%	1.57	− 0.15 (0.00)	25%	0.45	− 0.87 (0.00)
	Quintile 1		3.12	− 0.72 (0.00)		2.01	− 0.98 (0.00)
	Quintile 2		0.69	0.18 (0.05)		0.06	0.22 (0.50)
	Quintile 3		0.95	0.36 (0.00)		0.15	0.41 (0.03)
	Quintile 4		1.53	0.04 (0.50)		0.01	− 0.37 (0.69)
	Quintile 5		1.58	0.17 (0.00)		0.05	0.06 (0.83)
2019	Total	10%	2.60	− 0.07 (0.00)	25%	0.36	− 0.71 (0.00)
	Quintile 1		3.55	− 0.47 (0.00)		1.45	− 0.87 (0.00)
	Quintile 2		2.13	0.02 (0.70)		0.02	0.51 (0.33)
	Quintile 3		2.34	0.04 (0.43)		0.04	0.18 (0.63)
	Quintile 4		2.20	− 0.02 (0.72)		0.12	0.10 (0.62)
	Quintile 5		2.78	0.08 (0.05)		0.14	0.16 (0.35)
2020	Total	10%	1.32	− 0.32 (0.00)	25%	0.57	− 0.78 (0.00)
	Quintile 1		3.16	− 0.76 (0.00)		2.41	− 0.90 (0.00)
	Quintile 2		0.72	0.11 (0.23)		0.03	− 0.64 (0.18)
	Quintile 3		0.82	0.28 (0.00)		0.24	0.37 (0.01)
	Quintile 4		1.11	0.17 (0.01)		0.07	0.15 (0.56)
	Quintile 5		0.77	0.05 (0.47)		0.12	− 0.44 (0.02)
2021	Total	10%	1.24	− 0.05 (0.10)	25%	0.28	− 0.72 (0.00)
	Quintile 1		2.09	− 0.62 (0.00)		1.14	− 0.74 (0.00)
	Quintile 2		0.76	− 0.33 (0.00)		0.01	− 0.23 (0.77)
	Quintile 3		0.49	0.12 (0.25)		0.10	0.08 (0.74)
	Quintile 4		0.96	0.22 (0.00)		0.02	1.00 (0.03)
	Quintile 5		1.90	0.08 (0.07)		0.10	0.04 (0.82)

Table 4 presents key indicators of the distribution analysis with estimates of the Gini coefficient for incomes, concentration index for OOP payments, and Kakwani Index for assessing vertical inequality. Gini coefficient estimates vary around 0.28, indicating a relatively low level of income inequality. The concentration index also suggests a fairly uniform concentration of OOP expenses across all quintiles. The Kakwani Index was negative in 2018–2019 and positive in 2020 and 2021, but in all cases, it was close to zero, indicating a proportional system of OOP payments for healthcare.

**Table 4:** Distributional analyses of out-of-pocket payments.

Year		Gini index	Concentration index	Kakwani index
2018	Estimate	0.28087	0.27775	− 0.00312
	Std. Err	0.00093	0.00643	0.00778
	Z-value	302.13193	43.17905	− 0.40080
	P > Z	< 0.001	< 0.001	0.69
2019	Estimate	0.28159	0.27581	− 0.00578
	Std. Err	0.00086	0.00564	0.00683
	Z-value	326.90292	48.94122	− 0.84682
	P > Z	< 0.001	< 0.001	0.39710
2020	Estimate	0.27575	0.27837	0.00262
	Std. Err	0.00089	0.00679	0.00869
	Z-value	310.89191	40.99616	0.30094
	P > Z	< 0.001	< 0.001	0.76346
2021	Estimate	0.28552	0.29006	0.00454
	Std. Err	260.39783	0.00722	0.00900
	Z-value	0.00722	40.19884	0.50395
	P > Z	< 0.001	< 0.001	0.61430

**Figure 3:**
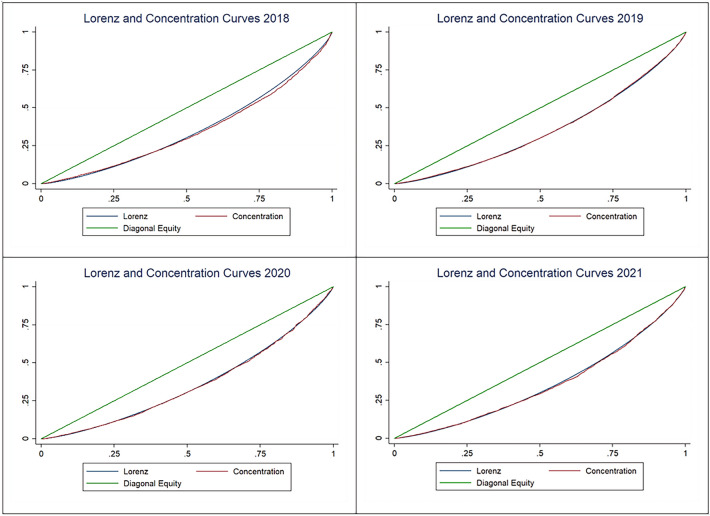
Lorenz and concentration curves.

A graphical representation of the indices from Table 4 is depicted in Fig. 3.

To assess the factors influencing the concentration index for OOP health expenditures, a regression of a RIF regression was employed (Table 5). The most significant factors throughout the study period include household size, the number of children in the household, as well as certain educational categories.

**Table 5:** RIF-I-OLS concentration index decomposition of healthcare OOP.

	2018		2019		2020		2021	
	Coef	Std. Error	Coef	Std. Error	Coef	Std. Error	Coef	Std. Error
Height	− 0.000	0.001	0.001	0.001	− 0.000	0.001	− 0.000	0.001
Household size	0.004	0.005	0.011**	0.005**	− 0.015**	0.006**	0.020**	0.006**
Number of children	− 0.026**	0.006**	− 0.023**	0.006**	0.002	0.007	0.018**	0.007**
Age	− 0.001**	0.001**	− 0.001**	0.000**	− 0.001	0.001	0.000	0.001
Gender = 2	− 0.005	0.014	− 0.007	0.013	− 0.002	0.016	0.017	0.016
No education	0.759*	0.432*	− 0.140	0.214	0.099	0.506	0.758**	0.282**
Preschool	Omitted	Omitted	0.218	0.427	Omitted	Omitted	0.833	0.720
Elementary	0.732*	0.395*	− 0.202	0.165	0.041	0.482	0.648**	0.229**
Basic secondary	0.761*	0.392*	− 0.187	0.157	0.123	0.479	0.613**	0.222**
Vocational secondary	0.740*	0.392*	− 0.223	0.156	0.050	0.478	− 0.705**	0.221**
Undergraduate	0.757*	0.392*	− 0.203	0.156	0.054	0.478	0.677**	0.221**
Graduate	1.651**	0.430**	Omitted	Omitted	0.306	0.525	Omitted	Omitted
Married	− 0.001	0.053	0.062	0.049	0.034	0.057	0.099*	0.056*
Divorced	− 0.062	0.057	0.072	0.053	0.018	0.061	0.182**	0.061**
Widow	− 0.014	0.058	0.072	0.054	0.061	0.062	0.095	0.063
Single	− 0.026	0.050	0.022	0.047	0.013	0.054	0.097*	0.053*
Constant	− 0.332	0.422	0.365	0.218*	0.314	0.508	1.004**	0.287**
N	31.058		30.938		30.962		30.711	

**p < 0.05, *p < 0.1

## Discussion

To the best of our knowledge, this is the first descriptive study identifying assess the progressivity of OOP payments for healthcare, factors influencing the inequality of healthcare OOP payments in Kazakhstan. We discovered a proportional system of payments OOP and a low incidence of CHE, especially during the Coronavirus disease 2019 (COVID-19) pandemic.

The issue of a high proportion of private expenditures in healthcare was initially highlighted in the healthcare development program “Densaulyk” for the years 2016–2019^27^. With the objective of mitigating financial risks arising from the escalating public and private healthcare expenditures, a phased implementation of social health insurance (SHI) was proposed^28^. The problem of the increasing private expenditures was further emphasized in the subsequent discontinued healthcare development program for 2020–2025, where this indicator served as a performance metric, indicating a reduction from 38.5% in 2018 to 26.9%^29^. Indeed, as indicated by the Global Health Expenditure Database, a reduction in OOP healthcare expenditures is observed, associated with the emergence of SHI in the financing structure and an increase in the share of other funding sources. This aligns with the decrease in CHE identified in our study^30^.

The obtained data of CHE are lower than WHO estimates for the 10% threshold: 2.61% in 2018, with a further increase in incidence to 3.72% in 2021. Perhaps this is due to differences in the assessment methodology, such as peculiarities of including certain age groups or features of including the household head for the AES assessment^31^.

Possibly, the decrease in overall spending on medical goods and services OOP in 2020–2021 could be related to enhancing public healthcare, bolstering health insurance, and increasing health financing are crucial steps for providing financial risk protection to households facing severe COVID-19, preventing catastrophic health expenditures^32^. The decrease in the incidence of CHE in 2020–2021 may be attributed to individuals’ reluctance to seek care for non-COVID-19-related conditions due to concerns about the potential impact of the virus. This was evidenced by the decrease in the number of visits to both private and public healthcare providers for primary healthcare services^33^.

Joseph Kutzin, in his publication, highlighted that equity or fairness in healthcare financing, where households contribute to the healthcare system based on their ability to pay, should constitute a crucial goal for advancing the objectives of UHC, according to the WHO^7^. Interestingly, OOP payments are regressive in nature^34^. For instance, in the province of Shiraz (Iran) in 2018, the Gini index for income stood at 0.297, the concentration index for OOP health expenditures was 0.174, and the Kakwani progressivity index calculated for OOP health expenditures amounted to − 0.123^35^. In a study conducted in Tanzania, the Gini index for income was 0.178, the concentration index for OOP expenditures was − 0.010, and the Kakwani index for OOP expenditures was − 0.187^36^. However, there are some studies, especially in developing countries, reporting that the Kakwani index is positive, corresponding to a progressive model. For example, in a review study on the measurement of progressivity, the authors compiled published Kakwani index data for low and middle-income countries. According to the authors, the most progressive healthcare financing systems were found in Bangladesh (0.210), Thailand (0.200), and Malaysia (0.180). Among the countries in the Central Asian and former Soviet Union region included in the review, Kyrgyzstan was the sole country, with a Kakwani Index of 0.010, indicating a proportional healthcare financing system in the country^37^. Our results also demonstrate a proportional system of OOP healthcare payments, with the Kakwani index showing positive values in 2020–2021. There are several explanations for this paradoxical system: firstly, in some countries, the poor cannot afford to spend money on healthcare and primarily rely on free government services, secondly, people with high incomes can afford more paid services, which need to be paid OOP^34^. The Sierra Leone case shows that rural residence and larger household sizes significantly influence the distribution of public healthcare benefits. Specifically, an increase in the proportion of rural households and households with 5–7 members leads to a higher concentration of benefits among certain income groups^38^.

According to data from the International Monetary Fund (IMF), the healthcare expenditure per capita in Kazakhstan is $12,970, as a percentage of GDP^39^. Current healthcare expenditures account for 3.7% of the GDP. In countries with a similar economic profile, the share of GDP allocated to healthcare spending is significantly higher: 7.39% in the Russian Federation (27% of which is OOP, 7.7% is CHE), 5.38% in China (34.4% of which is OOP, 24.33% is CHE), 10.55% in Montenegro (38% of which is OOP, 10.27% is CHE), 10% in Serbia (35.76% of which is OOP, 11.41% is CHE), and comparable figures in Malaysia are 4.38% (32% of which is OOP, 1.52% is CHE) and slightly progressive OOP expenditures scheme^26,27^. In the province of Shiraz (Iran) in 2018, CHE amounted to a substantial 16.48%. Renting accommodation, having a disabled member or a child under 5 years old in the household, and the absence of an extended insurance package increased the likelihood of experiencing catastrophic health expenditures^40^. The prevalence of CHE in Iran increased from 3.60% in 2013 to 3.95% in 2019. Rural populations consistently experienced a higher incidence of CHE compared to urban populations. Utilization of dental, outpatient, and inpatient care, as well as the presence of elderly household members, were associated with a higher probability of facing CHE^41^.

The obtained results reveal another paradoxical contradiction: despite a low share of healthcare expenditures in Kazakhstan’s GDP, the country exhibits a relatively high proportion of private healthcare expenditures. Despite this, Kazakhstan demonstrates a low incidence of catastrophic health expenditures and a fairly proportional payment system. This suggests that the high share of private payments does not worsen the financial resilience of the population. Particularly, the underprivileged segments of the population either do not spend significant amounts on healthcare, or conversely, government funding and SHI have an insufficient share in the financing structure.

In order to reduce the share of OOP payments in the healthcare system, the authors of the review recommend implementing strategies commonly utilized in both developed and developing countries. These strategies include conducting economic efficiency studies to determine threshold prices, incorporating dental care into medical insurance packages, government support for national health insurance programs, subsidy programs for diseases with high economic burdens, elimination of informal payments, free screening programs, performance-based payment systems, and the substitution of branded medications with generics^9^.

Limitations of our study may arise from our decision not to utilize total household expenditures as a measure of income. In developing nations, expenditures or consumption tend to be the preferred indicators. The rationale behind this preference in many developing countries is associated with inherent challenges in income measurement, such as the “seasonal variability of such income and a large share of income… derived from self-employment both in agriculture and other sectors”, aspects that households find difficult to account for as income. The substantial informal sector prevalent in developing countries further contributes to the unreliability of income data. Additionally, incomes might be concealed to evade taxation. Utilizing consumer expenditures of households, which reflect long-term welfare levels rather than immediate income, serves as a more dependable indicator of socioeconomic status. Furthermore, the unavailability of such data hindered our ability to conduct an analysis of impoverishment due to health expenditure^16,23^. Furthermore, our data from form D004 did not include information on food expenses, which prevented us from calculating the standard amount to cover basic needs for utilizing the approach to assess CHE as OOP expenses exceeding 40% after deducting the standard amount to cover basic needs. Another limitation of our study was the restricted number of factors considered, such as the number of individuals with disabilities and the number of individuals with chronic illnesses. An advantage of our study is that we conducted a comprehensive analysis of OOP healthcare expenditures, including FIA, using national-level data.

## Conclusion

OOP healthcare expenditures in Kazakhstan decreased during the pandemic in 2020–2021, both in absolute terms and as a percentage of total income. The incidence of CHE did not exceed 2.6% for the entire sample at the 10% threshold and was lower in 2020–2021. Also, during this period, there is a transition of the Kakwani index towards the positive side. The OOP payment system is characterized by proportionality throughout the study period. Household size, the number of children in the household, and certain educational categories were factors influencing the concentration index for OOP healthcare expenditures. The increase in state and other types of healthcare financing, quarantine restrictions, had an impact on reducing OOP payments for health goods and services.

## Data Availability

All data availability requests should be addressed to the corresponding author.
